# Establishment of the MethyLight Assay for Assessing Aging, Cigarette Smoking, and Alcohol Consumption

**DOI:** 10.1155/2015/451981

**Published:** 2015-10-22

**Authors:** Kosuke Endo, Jiawei Li, Michio Nakanishi, Takashi Asada, Masahiro Ikesue, Yoichi Goto, Yasue Fukushima, Naoharu Iwai

**Affiliations:** ^1^Department of Genomic Medicine, National Cerebral and Cardiovascular Center, 5-7-1 Fujishirodai, Suita, Osaka 565-8565, Japan; ^2^Department of Cardiovascular Medicine, National Cerebral and Cardiovascular Center, 5-7-1 Fujishirodai, Suita, Osaka 565-8565, Japan; ^3^Clinical Laboratory, National Cerebral and Cardiovascular Center, 5-7-1 Fujishirodai, Suita, Osaka 565-8565, Japan

## Abstract

The environmental factors such as aging, smoking, and alcohol consumption have been reported to influence DNA methylation (DNAm). However, the versatility of DNAm measurement by DNAm array systems is low in clinical use. Thus, we developed the MethyLight assay as a simple method to measure DNAm. In the present study, we isolated peripheral blood DNA from 33 healthy volunteers and selected cg25809905, cg02228185, and cg17861230 as aging, cg23576855 as smoking, and cg02583484 as alcohol consumption biomarkers. The predicted age by methylation rates of cg25809905 and cg17861230 significantly correlated with chronological age. In immortalized B-cells, DNAm rates of two sites showed a younger status than the chronological age of donor. On the other hand, the predicted age of the patients with myocardial infarction (MI) was not accelerated. The methylation rate of cg23576855 was able to discriminate the groups based on the smoking status. The DNAm rate of cg02583484 was reduced in subjects with habitual alcohol consumption compared to that of subjects without habitual alcohol consumption. In conclusion, our MethyLight assay system reconfirms that aging, smoking, and alcohol consumption influenced DNAm in peripheral blood in the Japanese. This MethyLight system will facilitate DNAm measurement in epidemiological and clinical studies.

## 1. Introduction

DNA methylation (DNAm) is a chemical modification underlying the epigenetic regulation of gene expression [1, 2]. Methylation of the cytosine residue in the CpG dinucleotide sites negatively regulates gene transcription through the recruitment of chromatin remodeling factors. Recently, it has been recognized that DNAm changes can be induced by lifestyle and environmental factors [3]. Therefore, the DNAm rate may represent a biological index of a lifetime of exposure to environmental factors, such as aging [4, 5], smoking [6–8], and alcohol consumption [9, 10].

Horvath reported that biological age could be predicted by DNAm of 353 CpG sites in multiple human tissues [11]. Afterwards, the difference between biological age and chronological age was used as an index of risk for age-related diseases [11, 12]. In addition, Weidner et al. reported that the chronological age could be tracked by the DNAm rate of peripheral blood DNA at just three CpG sites, located in the* ITGA2B*,* ASPA*, and* PDE4C* genes [13].

Moreover, a number of studies provide evidence that DNAm is associated with other lifestyle habits such as smoking and alcohol consumption. The methylation rates of cg23576855 and cg05573951 in* AHRR*, cg03636183 in* F2RL3*, and cg21566642 in* ALPPL2* are well known biomarkers for cigarette smoking [7, 8], and more than thirty CpG sites were reported as significantly associated probes in short-term alcohol dependence treatment programs [9].

DNAm patterns of candidate sites have been shown to serve as useful biomarkers for various situations. However, because DNAm analysis is mainly performed by DNA methylation array systems, the versatility of DNAm measurement is low in clinical use. Thus, the goal of the present study is to eliminate impediments to measure the DNAm rate of candidate sites. The MethyLight assay is a methylation-specific PCR-based technique, which is a quick and easy method for measuring DNAm, and has the potential to readily screen large numbers of samples [14]. In the present study, we focused on DNAm changes in the blood to establish a convenient assay system for epidemiological and clinical studies. We designed the TaqMan minor groove binder (MGB) probes of sites cg25809905 in* ITGA2B*, cg02228185 in* ASPA*, and cg17861230 in* PDE4C* for assessing chronological aging, cg23576855 in* AHRR* for assessing smoking, and cg02583484 in* HNRNPA1* for assessing alcohol consumption.

## 2. Materials and Methods

### 2.1. Preparation of Assay Template

Human reference DNA that was isolated from anonymous peripheral blood was purchased from Clontech-TAKARA (CA, USA). DNA was bisulfite-converted using a MethylEdge Bisulfite Conversion System (Promega, WI, USA), following the manufacturer's recommendations. Fragments including the target site were PCR-amplified from the bisulfite-converted DNA using primer sets (Table 1) with EpiTaq HS DNA polymerase (TAKARA, Shiga, Japan) as described previously [15]. The PCR product was cloned into the pTA2 vector using a TArget Clone Plus cloning kit (TOYOBO, Osaka, Japan). DNA sequencing was performed on a Genetic Analyzer 3130 (Applied Biosystems, CA, USA) to confirm the cloned sequence by using the M13F and M13R primers.

### 2.2. Development of the MethyLight Assay System

The methylated and unmethylated DNA-specific TaqMan MGB probes (Table 1) were designed to detect methylated and unmethylated cytosine bases, respectively. A panel of eleven DNA standard samples was prepared by combining the following proportions of plasmid DNA with methylated and unmethylated types of targets: 0%, 10%, 20%, 30%, 40%, 50%, 60%, 70%, 80%, 90%, and 100% methylated DNA. The MethyLight assay was performed on DNA standards and a standard curve was drawn using methylation scores, which were calculated using the C_p_ values of the FAM-labeled probe for methylated DNA (C_pFAM_) and the VIC-labeled probe for unmethylated DNA (C_pVIC_) with the following equation:

(1) $$\begin{array}{c} \text{Methylation score}=\frac{2^{\left(35\text{-}{\text{C}}_{\text{pFAM}}\right)}}{\left(2^{\left(35\text{-}{\text{C}}_{\text{pFAM}}\right)}+2^{\left(35\text{-}{\text{C}}_{\text{pVIC}}\right)}\right)}. \end{array}$$

The assay was completed with a 7500 Real-Time PCR System (Applied Biosystems). Each 20 *μ*L of reaction mixture contained 20 fg of bisulfite-treated template DNA, 10 *μ*L of Universal MasterMix (Applied Biosystems), 100 nM of TaqMan MGB probes, and 10 *μ*M of TaqMan primers (Table 1). Thermal cycling was initiated with an enzyme activation step of 10 min at 95°C, followed by 40 cycles of the annealing step: 10 min for the first cycle and 1 min for the following 39 cycles at temperatures corresponding to each primer set (Table 1).

### 2.3. MethyLight Assay for Human Samples

Peripheral blood samples were collected from 33 healthy donors and 7 patients with myocardial infarction (MI) in tubes containing ethylenediaminetetraacetic acid (EDTA) as an anticoagulant. In addition, hair and oral mucosa were collected from some donors for preliminary study. In the part of samples, peripheral mononuclear cells and granulocytes were separated from the blood samples using the Lymphocyte Separation Solution (Nacalai Tesque, Kyoto, Japan). DNA samples were extracted using a DNeasy Blood & Tissue Kit (QIAGEN, CA, USA), according to the manufacturer's instructions. Hundred DNA samples (age groups 20–29, 30–39, 40–49, 30–39, and 40–49 years; *N* = 10 in each group) derived from immortalized Japanese B-cell lines were obtained from the National Institutes of Biomedical Innovation, Health, and Nutrition. The DNA samples were bisulfite-converted using the MethylEdge Bisulfite Conversion System. About 10 ng of human DNA sample was used for the MethyLight assay. Assays were performed in duplicate and the methylation score was calculated as described above. The standard curve was drawn and the coefficient of variation was calculated for each run. The methylation rates of human samples were estimated using the standard curve.

### 2.4. Ethical Statement

This study was approved by the Ethics Committee of the National Cardiovascular Center and performed in accordance with the Code of Ethics of the World Medical Association, and all subjects signed informed consent. Some physiological (sex, age, body weight, and height) and lifestyle (smoking history and alcohol consumption) data were obtained from interviews. Subject characteristics are shown in Table 2.

### 2.5. Statistical Analysis

Data are presented as mean ± SD. Statistical analysis was performed by regression analysis, ROC analysis, ANCOVA, ANOVA, and Tukey's HSD test using the JMP statistical analysis package (SAS Institute, NC, USA).

## 3. Results

### 3.1. Age Prediction

**Figure 1:**
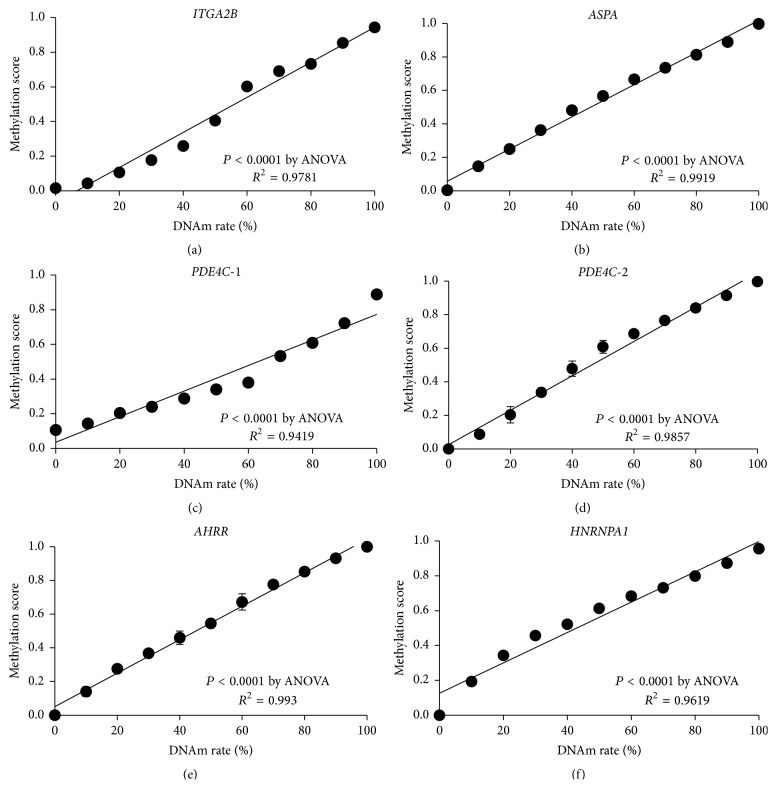
Development of the MGB probes for the MethyLight assay. The standard curves of four DNAm sites (cg25809905 in* ITGA2B* (a), cg02228185 in* ASPA* (b), cg17861230 in* PDE4C *(c, d)), cg23576855 in* AHRR *(e), and cg02583484 in* HNRNPA1* (f) were able to detect 10% change of DNAm rate of template mixture. Assays were performed in triplicate, and each 10% difference could be statistically discriminated (*P* < 0.05, by Bonferroni correction).

**Figure 2:**
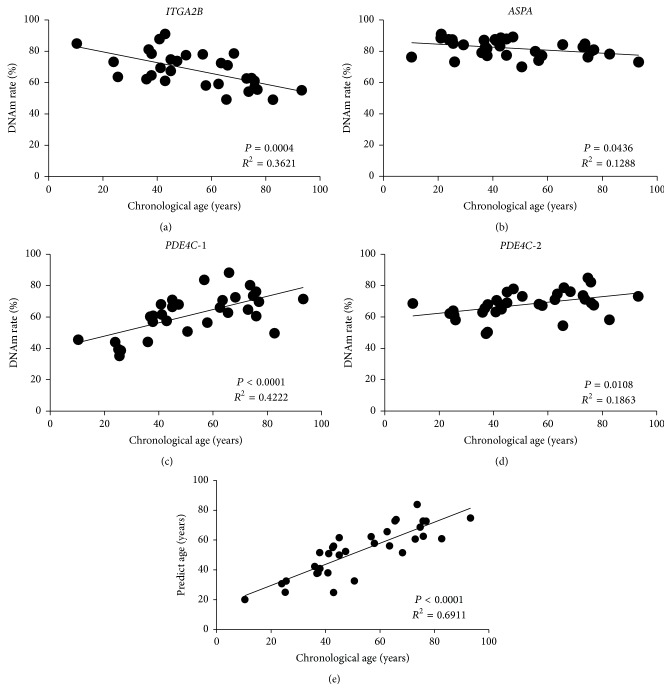
DNAm rates of four DNAm sites measured by the MethyLight assay. DNAm rates of cg25809905 in* ITGA2B* (a), cg02228185 in* ASPA* (b), and cg17861230 in* PDE4C *(c, d) were measured using the MethyLight assay. DNAm rates correlated to the chronological age. As a result, the predicted age was estimated from cg25809905 in* ITGA2B* and cg17861230 in* PDE4C*-1 (e).

The MethyLight probes for four sites (cg25809905, cg02228185, and two sites in cg17861230) were able to detect 10% change in the DNAm rate of template mixture (Figures 1(a)–1(d)). Based on these results (Table S1, in Supplementary Material available online at http://dx.doi.org/10.1155/2015/451981), the reproducibility of this assay appears to be very high. As a preliminary study, using the DNA samples derived from blood, hair, and oral mucosa, the methylation rate of cg25809905 in* ITGA2B* was measured. As a result, cg25809905 in* ITGA2B* in hair and oral mucosa was methylated approximately, and the correlation with the age was absent (Figure  S1). Therefore, it was studied with a focus on blood. The DNAm rate of all 4 candidate sites correlated to chronological age (cg25809905 in* ITGA2B*: *r* ^2^ = 0.362, *P* = 0.0004; cg02228185 in* ASPA*: *r* ^2^ = 0.129, *P* = 0.0436; cg17861230 in* PDE4C *(*PDE4C-1*): *r* ^2^ = 0.422, *P* < 0.0001; cg17861230 in* PDE4C* (*PDE4C-2*): *r* ^2^ = 0.186, *P* = 0.0108; Figures 2(a)–2(d)). To define the age predictor, the DNAm rates at four sites, sex, BMI, smoking history, and alcohol drinking history were treated as independent variables in a multiple regression analysis. *F*-tests were used to assess the model. Only two sites, cg25809905 (*ITGA2B*) and cg17861230 (*PDE4C*-1), were significant independent variables (Table S2). The predicted age was estimated from the following formula:

(2) Age prediction=65.76+0.92×DNAmPDE4C-1−1.03×DNAmITGA2B.

The predicted age correlated to the chronological age (Figure 2(e); *r* ^2^ = 0.6911, *P* < 0.0001).

**Figure 3:**
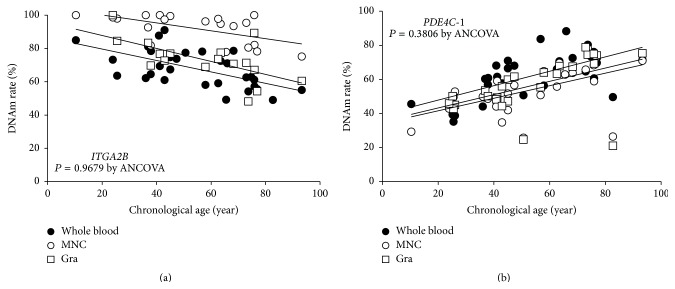
DNAm rates of peripheral blood cells. To evaluate the influences of cell composition, the DNAm rates of cg25809905 in* ITGA2B* (a) and cg17861230 in* PDE4C*-1 (b) were assessed in peripheral mononuclear cells and granulocytes. The DNAm rates were assessed by ANCOVA and showed no significant difference between whole blood (closed circle), peripheral mononuclear cells (open circle: MNC), and granulocytes (open square: Gra) ((a) *P* = 0.9679, (b) *P* = 0.3806).

The epigenetic age predictions might be influenced by differences of the cellular composition in blood, because the blood cellular composition is affected by aging [16]. Thus, we assessed the DNAm rate of cg25809905 (*ITGA2B*) and cg17861230 (*PDE4C*-1) in peripheral mononuclear cells and granulocytes in the part of samples. Our results indicate that the DNAm sites of these two loci were not cell-type-dependent (Figures 3(a) and 3(b), *P* = 0.9679 for cg25809905 and *P* = 0.3806 for cg17861230).

**Figure 4:**
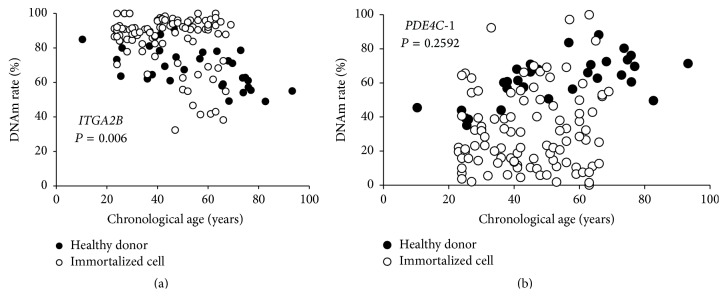
DNAm rates of immortalized human B-cells. DNAm rates of cg25809905 in* ITGA2B* (a) and cg17861230 in* PDE4C*-1 (b) were measured in DNA derived from immortalized human B-cells (open circle). DNAm rates did not correlate with the chronological age. The closed circles represent results of 33 healthy donors.

Subsequently, DNAm rates of cg25809905 in* ITGA2B* and cg17861230 in PDE4C-1 were measured and predicted age was estimated for DNA derived from immortalized human B-cells. The results indicated that DNAm rate of cg25809905 in* ITGA2B* was correlated with the age of donor (Figure 4). However, the age prediction using the previous formula has been impossible. DNAm status was considered to change according to the immortalization. In addition, although individual differences were large, plots of the methylation scores versus the age of the donor tended to show younger DNAm status for immortalized samples compared to collected nonimmortalized samples.

**Figure 5:**
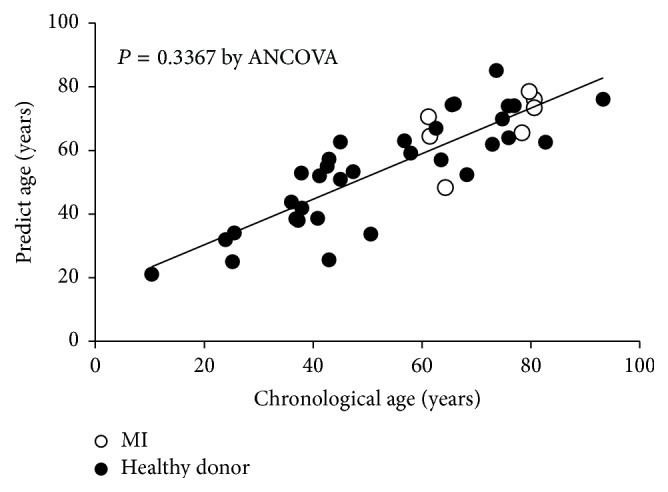
Predicted age of patients with myocardial infarction. The predicted age of patients with MI (open circle) was calculated and compared with that of healthy donors (closed circle). The DNAm-predicted age was not affected by MI, and the predicted age of both healthy donors and patients with MI could be estimated by the same equation.

Furthermore, the predicted age of MI patients was calculated by an equation based on data from healthy donors, and we assessed whether MI accelerated predicted age. The results indicated that predicted age was not affected by MI; the predicted age of both healthy donors and patients with MI could be estimated by the same equation (Figure 5).

### 3.2. DNA Methylation Changes by Smoking

**Figure 6:**
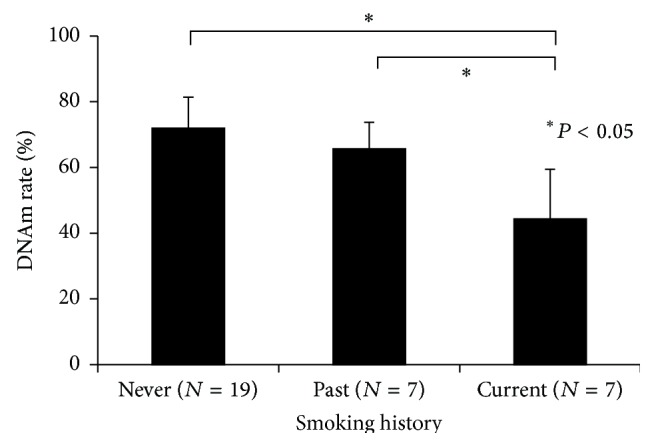
Assessment of the detection of smoking history using the MethyLight assay. Cigarette smoking accelerated demethylation of cg23576855 in* AHRR*. Thus, the MethyLight assay was able to categorize the never, past, and current smoker groups (*P* < 0.0001 by ANOVA). Subsequently, differences among groups were assessed by ANOVA followed by Turkey HSD test. ^*∗*^ *P* < 0.05. Error bars, mean ± SD.

The probe for cg23576855 (*AHRR*) was able to detect a 10% change in the DNAm rate of the DNA standard (Figure 1(e) and Table S1). In the present study, the subjects were categorized into three groups based on smoking history: never smoker (*N* = 19), past smoker (more than two months of prohibition of smoking, *N* = 7), and current smoker (average cigarettes/day: 22 ± 8, *N* = 7). Based on a multiple regression analysis, the DNAm rate of cg23576855 was only correlated with smoking history and not with other physiological parameters (Table S2). The methylation rate of cg23576855 in* AHRR* was significantly different between the current and other two groups (Figure 6, *P* < 0.0001 by ANOVA, never: 72.0 ± 9.5, past: 65.8 ± 8.0, current: 44.4 ± 15.1). Smoking has accelerated the demethylation of cg23576855 in* AHRR*. Based on the ROC analysis, the AUC for the DNAm rate was 0.955 for current smoking. The DNAm rate cutoff point was 58.96% for current smoking (Figure S2 A). Moreover, the methylation rate of cg23576855 in* AHRR* has been restored, to some extent, in past smokers (Figure 6). In current smoking group, the DNAm rate of cg23576855 was not correlated to the frequency of the cigarette smoking (data not shown).

### 3.3. DNA Methylation Changes by Alcohol Consumption

**Figure 7:**
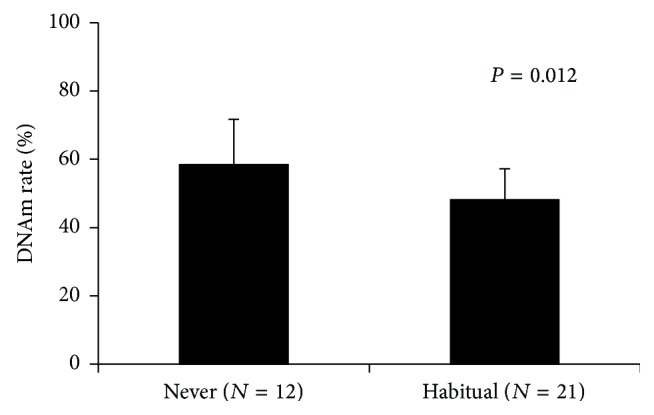
Assessment of the detection of habitual alcohol drinking using the MethyLight assay. The subjects were divided into two groups: with and without habitual alcohol drinking. The DNAm rate of cg02583484 in* HNRNPA1* was decreased by habitual alcohol intake (*P* = 0.012 by Student's *t*-test). No = the group without habitual alcohol intake; Yes = the group with habitual alcohol intake

The probe for cg02583484 in* HNRNPA1* was able to detect a 10% change in the DNAm rate of the DNA standard (Figure 1(f) and Table S1). The subjects were divided into two groups (no one has belonged to occasional drinking group): never (*N* = 12) and habitual alcohol drinking (*N* = 21) (Table 2). Based on the multiple regression analysis, the DNAm rate of cg02583484 was only correlated with drinking history and not with other physiological parameters (Table S2). The group with habitual alcohol drinking had a lower cg02583484 DNAm rate in* HNRNPA1* than the DNAm of the group without habitual alcohol consumption (Figure 7, *P* = 0.012 by Student's *t*-test, never: 58.5 ± 13.2, habitual: 48.1 ± 9.1). Based on the ROC analysis, the AUC for the DNAm rate was 0.746 for current smoking. The DNAm rate cutoff point was 56.69% for current smoking (Figure S2 B). However, the DNAm value of cg02583484 was not correlated to alcohol consumption in the habitual drinking group (data not shown).

## 4. Discussion

Nowadays, various environmental factors have been considered to contribute to genetic and epigenetic modifications and influence phenotypic variations. Aging, smoking, and alcohol consumption have been reported to influence the methylation rates of many CpG islands in peripheral blood genomic DNA, as described in Introduction.

The purpose of the present study is to reconfirm that aging, smoking, and alcohol consumption influence the methylation rates of several sites of CpG islands in peripheral blood genomic DNA in the Japanese and to establish a convenient method for assessing DNAm in several candidate sites, using MethyLight assay system.

The MethyLight assay demonstrated an extended quantitative range as compared to methylation-sensitive restriction enzyme qPCR or methylation-dependent enzyme qPCR in previous studies [17, 18]. On the other hand, although bisulfite amplicon next-generation sequencing (NGS) has the advantage that it is possible to analyze DNAm comprehensively, a systematic bias to overestimate the rate of DNAm is evident [19]. The MethyLight probes in the present study were able to detect 10% change of DNAm rate of template mixture (Figure 1), and it was expected to be extremely accurate. The MethyLight probes and primers used in the study have different melting temperature (*T*_*m*_) values and the assays were performed at different annealing temperatures according to the targets (Table 1). Therefore it is expected that the MethyLight assay would show higher accuracy than Illumina Infinium Methylation assay in which hybridization temperature is at one temperature. In the present study, using the MethyLight assay, we have constructed aging and lifestyle (smoking and alcohol) biomarkers for the first time in a Japanese population. We constructed the MethyLight assay system for four CpG sites, located in genes* ITGA2B*,* ASPA,* and* PDE4C*, which were found by Weidner et al. based on comprehensive analyses of DNAm profiles and bisulfite pyrosequencing [13]. Subsequently, we determined the epigenetic age using our assay system and it was able to estimate predicted age from only two CpG sites: cg25809905 (*ITGA2B*) and cg17861230 (*PDE4C-1*). This difference between our result and previous studies might be due to the race of the subjects; thus further studies on large numbers of subjects are required to clarify the causes.

The epigenetic age acceleration derived by DNAm is a heritable trait that predicts mortality independently of health status, lifestyle factors, and known genetic factors [20]. It was reported that the “Horvath age,” or epigenetic clock, was accelerated in HIV-1-infected adults [12] and Down syndrome, which entails an increased risk typically associated with older age [21]. In present study, we assessed changes in predicted age obtained from the DNAm status of cg25809905 (*ITGA2B*) and cg17861230 (*PDE4C*-1) in Epstein-Barr virus- (EBV-) induced immortalized human B-cells and patients with MI. In immortalized human B-cells, individual differences appear large and DNAm rates of two sites showed a younger status than the chronological age of donor. This was probably due to the process of immortalization. B cells were transformed into actively proliferating B-lymphoblastoid cell lines (LCLs) by infection with EBV. Most LCLs from normal individuals are mortal because their telomeres shorten upon division. Some LCLs are truly immortalized by accidentally developing strong telomerase activity and chromosomal rearrangement [22]. Therefore, the DNAm status in immortalized B-cells might be rejuvenated by acquisition of proliferative capacity, and individual differences in the DNAm status might be caused by chromosomal rearrangement. On the other hand, the predicted age of MI patients was not accelerated by MI. The seven patients with MI did not have chronic heart failure and their prognosis was relatively stable. Thus, the epigenetic clock may not have been accelerated. To use the epigenetic age estimated from DNAm as prognostic factor in future epidemiological studies, it is necessary to observe changes in epigenetic age in more diseases. Recently, it was reported that DNA methylation status associated with T cell mediated immune response in CD8+ T cells was correlated with age and there was strong inverse correlation between DNA methylation and the gene expression [23]. Thus, it was suggested that the reduction of the biological function of a living body due to aging might be estimated by DNA methylation status.

The DNAm rates of cg23576855 in* AHRR* and cg02583484 in* HNRNPA1* were able to discriminate smoking history and alcohol consumption, respectively. Notably, the DNAm rate of cg23576855 in* AHRR* was significantly reduced in the currently smoking group. Philibert et al. reported that cg23576855 in* AHRR* was a site for a CG/CA SNP (rs6869832) [24] and its minor allele frequency was 0.0611 in the NCBI database. If this mutation is present, MethyLight probes used in the present study may not detect the DNAm rate of the target site. However, DNAm rates could be measured in all samples in this study. In addition, rs6869832 was not reported in a SNP database in Japanese population (Human Genetic Variation Browser: http://www.genome.med.kyoto-u.ac.jp/SnpDB/); therefore, the effect of rs6869832 can be ignored and the measurement of cg23576855 has high accuracy.* AHRR* is a feedback modulator by repressing AhR-dependent gene expression and is involved in the regulation of cell growth and differentiation [25]. Exposure to cigarette smoke induces polycyclic aromatic hydrocarbons (PAHs) that trigger the AhR signaling pathway [26, 27]. It is suggested that the induction of* AHRR* expression by smoking is through demethylation. In this sense, the reduction of methylation rate of cg23576855 in* AHRR* might have a biological significance.

The assessment of cg23576855 in* AHRR* methylation might be useful in epidemiological studies to confirm smoking status and to assess indirect passive smoking status. In addition, the assessment of the methylation rate of cg02583484 in* HNRNPA1* might be useful in epidemiological studies to confirm alcohol consumption, which might be unreliable based on self-assessment. Although it will be ideal to detect the frequency of cigarette smoking and alcohol drinking more clearly, the DNAm rates of* AHRR* and* HNRNPA1 *have not correlated to the frequency of smoking and alcohol drinking in present study. Thus, further studies on large numbers of subjects and accurate information are required for precise assessment.

Many biomarkers used to detect smoking or alcohol exposure have some limitations. For example, a half-life of cotinine, a well-validated biomarker for cigarette smoking, is 16 hours [28, 29]. This is a significant limitation for the assessment of lifestyle and/or diseases with long latency periods. Additionally, fatty-acid ethyl esters, ethyl glucuronide, and phosphatidylethanol are useful biomarkers for alcohol consumption. However, identification of these biomarkers requires gas or liquid chromatography techniques [30, 31]. Therefore, they are unsuitable for epidemiologic studies in which limited samples were conserved. In contrast, epigenetic modifications, such as DNAm and histone modification, can be biomarkers that overcome these limitations. Although further analyses are needed, DNAm analysis has the potential to monitor the diseases and/or lifestyles that result in no differences in protein and mRNA levels.

In conclusion, we identified biomarkers for aging, smoking history, and alcohol consumption using the MethyLight assay. By our assay system, when including data of exercise habit, it may be possible to see the influence of exercise on biological age. Similarly, when including the smoking status of the family, it may be possible to index passive smoking. MethyLight achieves low-cost and broad application status for analysis of a large number of samples. Therefore, it can be expected that this technique enables reasonable and convenient DNAm measurement in epidemiological studies.

## Supplementary Material

Establishment of the MethyLight assay for assessing aging, cigarette smoking, and alcohol consumption.

## Figures and Tables

**Table 1:** Primer set and probes.

Illumina ID	Symbol	PCR primer set	TaqMan primer set	*T*_*m*_ value of primer [°C]	TaqMan MGB probe	*T*_*m*_ value of probe [°C]	Temp [°C]
cg25809905	*ITGA2B*	F: GATTTGATTTTGGTTGGGGGTTTTG	F: GTTTAGGGGAGTTTTTTTTGATT	F: 53.9	Me: TGGTTG**C**GTGGGT	Me: 62.0	58
		R: CTCTAAAACTATAACAAAAAACCTTACTCCC	R: CAAAAATAAACAATATACTCAATACTATACCT	R: 53.7	deMe: TTGGTTG**T**GTGGGTT	deMe: 63.0	

cg02228185	*ASPA*	F: GAATTGTAGAAATTAGATAAAAATTATTTGGTG	F: TGGAGTATTTTTGGTTAAGTATTGG	F: 54.7	Me: AGAATGG**C**GTTGAGAT	Me: 64.0	60
		R: CACAATCAATATATCTAATACACTTCTTCACTACTC	R: AATTTTACCTCCAACCCTATTCTC	R: 55.0	deMe: AGAATGG**T**GTTGAGATT	deMe: 65.0	

cg17861230^*∗*^	*PDE4C*	F: GGTGAAGGGGTAGAGGTTTGTAG R: ACAACTTCCTAAACTCCAACCA	PDE4C-1		PDE4C-1		61
			F: GAGTTGTTTTTGTGGTTGTTATAGT	F: 52.6	Me: TTAGAGTTT**C**GAAGTATTT	Me: 66.0	
			R: TATTAAATACAAATAAAACACCAAAATT	R: 52.8	deMe: ATTAGAGTTT**T**GAAGTATTT	deMe: 65.0	
			PDE4C-2		PDE4C-2		
			F: TGTTTTTGTGGTTGTTATAGTATGATTAGAGTTT	F: 60.1	Me: TTGTGG**C**GGTAAT	Me: 62.0	
			R: CAAATAAAAACACTATTAAATACAAATAAAACACCA	R: 60.3	deMe: TTTGTGG**T**GGTAATT	deMe: 63.0	

cg23576855	*AHRR*	F: GGTGGATGGTTGGTAATGGTTT	F: GTGTTGGTAGGATATAGGGGTTGTTTA	F: 58.7	Me: TTATAGATT**C**GGGTTTAGG	Me: 65.0	60
		R: CCCAACCAAATACAAAACAAAACC	R: CAAAAATAAACAATCCCCACAACTAA	R: 58.4	deMe: TTATAGATT**T**GGGTTTAGG	deMe: 64.0	

cg02583484	*HNRNPA1*	F: TGGATAGGGTTATGGAAATTAGGGTAG	F: TTATGATAGTTATAATAATGGAGGTGGAGG	F: 58.4	Me: TTTTGG**C**GGTGGTA	Me: 61.0	61
		R: TCAACAACACCAAAACCCAAACT	R: ACACCAAATACTTAAATCACTAAATACCTACC	R: 58.2	deMe: GTTTTGG**T**GGTGGTA	deMe: 61.0	

The underlined and bold letters indicate the positions of methylation/unmethylation site.

F: forward primer, R: reverse primer, Me: probe for methylated site with FAM, and deMe: probe for unmethylated site with VIC.

^*∗*^cg17891230 has two methylated sites. PDE4C-1: upstream site; PDE4C-2: downstream site.

**Table 2:** Subject characteristics.

	Healthy donors	Patients with MI
Male/female	22/11	4/3
Age, M/F (years)	52.8 ± 19.2/56.0 ± 20.0	71.6 ± 10.4/73.1 ± 10.2
Height, M/F (cm)	167.4 ± 10.9/156.9 ± 4.9	162.8 ± 5.0/148.7 ± 6.0
Weight, M/F (kg)	66.0 ± 11.7/50.6 ± 6.0	62.1 ± 8.3/57.7 ± 13.6
BMI, M/F	23.3 ± 2.5/20.6 ± 2.4	23.6 ± 4.4/26.0 ± 4.9
Smoking history, M/F		
Never	10/9	4/3
Past	6/1	0/0
Current	6/1	0/0
Habitual alcohol drinking, M/F		
Never	8/4	0/0
Occasional	0/0	0/0
Habitual	14/7	0/0

Values are expressed as mean ± SD.

Smoking history categories: never, past smoker (more than two months without smoking), and current smoker (average cigarettes/day: 22 ± 8).

Alcohol drinking history categories: never drinking, occasional drinking (less than 40 g/week of alcohol consumption), and habitual drinking (more than 40 g/week of alcohol consumption).
