# Supplementary material for: Establishment of the MethyLight Assay for Assessing Aging, Cigarette Smoking, and Alcohol Consumption
Source: Biomed Res Int. 2015 Oct 22;2015:451981. doi: 10.1155/2015/451981 (PMC4633697; doi:10.1155/2015/451981)
Supplement: Supplementary file 1 — Establishment of the MethyLight assay for assessing aging, cigarette smoking, and alcohol consumption. [file 451981.f1.pdf]

Supplementary materials

Establishment of the MethyLight assay for assessing aging, cigarette smoking, and alcohol consumption

Kosuke Endo[1*], Jiawei Li[1*], Michio Nakanishi[2], Takashi Asada[3], Masahiro Ikesue[1], Yoichi Goto[2], Yasue Fukushima[1], and Naoharu Iwai[1]

Department of Genomic Medicine[1], Department of Cardiovascular Medicine[2], Clinical Laboratory[3], National Cerebral and Cardiovascular Center, 5-7-1 Fujishirodai, Suita, Osaka 565-8565, Japan

*These authors contributed equally to this work

Address correspondence to:

Kosuke Endo, PhD

Department of Genomic Medicine, National Cerebral and Cardiovascular Center

5-7-1 Fujishirodai, Suita, Osaka 565-8565, Japan

Tel: +81-6-6833-5012 Fax: +81-6-6835-2088

E-mail: ko-end@ncvc.go.jp

Evaluation of the reproducibility of the MethyLight assay

A panel of eleven DNA standard samples was prepared by combining the following proportions of plasmid DNA with methylated and unmethylated types of targets: 0%, 10%, 20%, 30%, 40%, 50%, 60%, 70%, 80%, 90%, and 100% methylated DNA. The MethyLight assay was performed in triplicate. The raw data of the Cp values were shown in Table S1.

Table S1 Data points of the Cp values of DNA standard

	Methylation % of DNA std	Cp value of Me run1	Cp value of Me run2	Cp value of Me run3	Cp value of deMe run1	Cp value of deMe run2	Cp value of deMe run3	Avg of DNAm score
	0	30.93	31.51	31.17	25.32	25.03	25.03	0.02
	10	30.30	30.00	29.89	25.69	25.55	25.51	0.04
	20	29.59	29.56	29.71	26.66	26.35	26.60	0.11
	30	28.65	28.66	28.71	26.50	26.40	26.46	0.18
cg25809905	40	27.08	27.23	27.40	25.62	25.75	25.77	0.26
ITGA2B	50	27.56	27.46	27.51	27.02	26.95	26.92	0.41
	60	27.02	26.93	26.99	27.56	27.66	27.51	0.60
	70	26.62	26.58	26.68	27.77	27.78	27.81	0.69
	80	26.74	26.68	26.80	28.17	28.20	28.21	0.73
	90	26.20	26.11	26.09	28.62	28.67	28.73	0.85
	100	25.79	25.74	25.74	29.78	29.86	29.81	0.94
	0	37.73	32.60	34.70	25.50	25.57	25.50	0.00
	10	28.38	28.51	28.46	25.82	25.99	25.89	0.15
	20	27.55	27.74	27.48	26.06	25.95	25.97	0.25
	30	27.02	27.22	27.27	26.20	26.52	26.36	0.36
cg02228185	40	26.50	26.72	26.61	26.22	26.72	26.56	0.48
ASPA	50	26.07	26.04	25.75	26.34	26.40	26.28	0.57
	60	25.70	26.19	26.09	26.79	27.19	26.99	0.67
	70	25.36	25.85	25.63	26.88	27.38	27.01	0.74
	80	25.18	25.55	25.34	27.28	27.72	27.43	0.81
	90	24.91	25.28	24.92	28.01	28.29	27.87	0.89
	100	25.70	25.67	25.50	38.51	38.84	32.67	1.00
	0	32.49	32.62	32.52	29.49	29.46	29.47	0.11
	10	32.26	32.24	32.28	29.65	29.69	29.66	0.14
	20	31.80	31.68	31.73	29.77	29.77	29.80	0.20
	30	31.41	31.43	31.45	29.72	29.78	29.80	0.24
cg17861230	40	31.28	31.23	31.26	29.92	29.97	29.96	0.29
PDE4C-1	50	31.01	31.10	30.94	30.06	30.06	30.06	0.34
	60	30.90	30.89	30.90	30.19	30.18	30.20	0.38
	70	30.57	30.56	30.55	30.75	30.75	30.75	0.53
	80	31.11	30.86	30.90	31.61	31.59	31.60	0.61
	90	31.10	30.98	30.91	32.37	32.41	32.37	0.72
	100	31.37	30.81	30.85	33.99	34.04	34.04	0.89
	0	50.00	50.00	50.00	24.50	24.46	24.37	0.00
	10	27.99	28.09	27.61	24.69	24.47	24.31	0.09
	20	26.40	26.40	25.78	23.93	24.45	24.19	0.20
	30	25.38	25.21	25.29	24.25	24.41	24.28	0.34
cg17861230	40	24.54	25.12	24.86	24.47	24.71	24.97	0.48
PDE4C-2	50	24.50	24.02	24.22	24.89	24.70	25.06	0.61
	60	24.05	23.73	24.03	25.18	24.86	25.17	0.69
	70	23.94	23.58	23.75	25.66	25.27	25.47	0.77
	80	23.54	23.41	23.34	25.93	25.79	25.74	0.84
	90	23.52	23.40	23.44	26.94	26.89	26.82	0.91
	100	20.81	20.75	20.41	28.98	28.96	28.96	1.00
	0	40.00	40.00	40.00	28.56	28.52	28.50	0.00
	10	31.56	31.07	31.60	28.84	28.80	28.70	0.14
	20	30.34	30.87	30.51	28.85	29.51	29.18	0.28
	30	29.52	29.56	29.22	28.87	28.80	28.29	0.37
cg23576855	40	29.85	29.47	29.64	29.36	29.31	29.59	0.46
AHRR	50	29.55	28.89	29.15	29.88	29.15	29.33	0.54
	60	29.09	28.88	28.99	30.01	29.68	30.41	0.67
	70	29.34	29.32	29.29	31.23	31.08	31.01	0.78
	80	28.80	28.70	28.73	31.33	31.24	31.28	0.85
	90	27.80	27.68	27.65	31.60	31.42	31.40	0.93
	100	26.06	26.26	26.13	38.24	38.39	38.60	1.00
	0	40.00	40.00	40.00	22.04	22.11	21.97	0.00
	10	24.63	24.45	24.51	22.54	22.37	22.49	0.19
	20	23.60	23.60	23.62	22.68	22.63	22.71	0.34
	30	23.38	23.19	23.23	23.05	22.90	23.11	0.46
cg02583484	40	22.82	22.72	22.82	22.91	22.98	22.85	0.52
HNRNPA1	50	22.37	22.41	22.36	23.08	23.06	23.01	0.61
	60	22.91	22.58	22.66	23.96	23.72	23.80	0.68
	70	22.02	22.22	22.17	23.52	23.62	23.61	0.73
	80	22.73	22.40	22.17	24.69	24.31	24.28	0.80
	90	22.23	21.76	21.94	24.80	24.73	24.77	0.87
	100	21.80	21.74	21.73	26.04	26.20	26.30	0.96

The multiple regression analysis using each factors.

To define the age predictor, the DNAm rates at four sites, sex, BMI, smoking history, and alcohol drinking history were treated as independent variables in a multiple regression analysis.

Furthermore, to evaluate the effect on AHRR and HNRNPA1 methylation status, age, sex, BMI, smoking history, and alcohol drinking history were treated as independent variables in a multiple regression analysis. The *p*-values were shown in Table S2.

Table S2 The results of the multiple regression analysis

	independent variable	*P* value
age	PDE4C1	0.036
	ITGA2B	<.0001
	ASPA	0.419
	PDE4C2	0.403
	sex	0.900
	BMI	0.790
	smoking	0.812
	alcohol	0.630
AHRR	age	0.164
	sex	0.564
	BMI	0.323
	smoking	<.0001
	alcohol	0.747
HNRNPA1	age	0.520
	sex	0.230
	BMI	0.278
	smoking	0.722
	alcohol	0.023

The measurement of the DNAm rate in genomic DNA derived from hair and oral mucosa

(A)

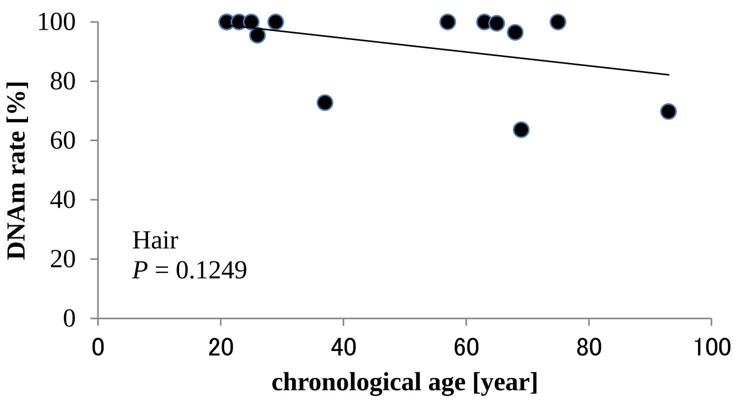

(B)

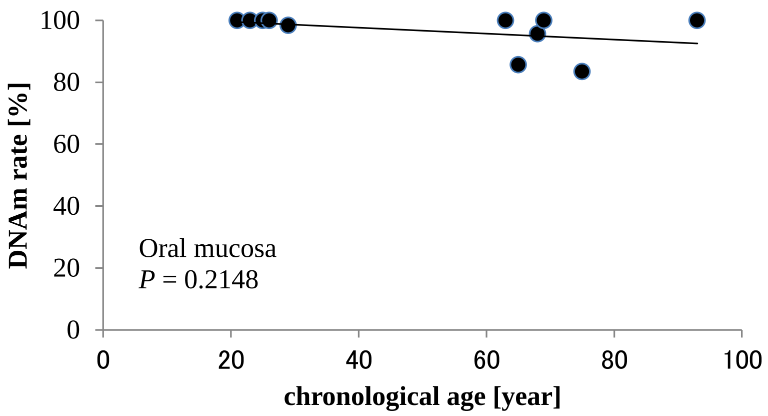

Figure S1 The methylation rate of cg25809905 in *ITGA2B* in hair and oral mucosa

As a preliminary study, the methylation rate of cg25809905 in *ITGA2B* was measured in some DNA samples derived from hair (A) and oral mucosa (B). As a result, cg25809905

in *ITGA2B* in hair and oral mucosa was methylated approximately, and the correlation with the age was absent.

ROC curve analysis for smoking history and alcohol drinking

(A)

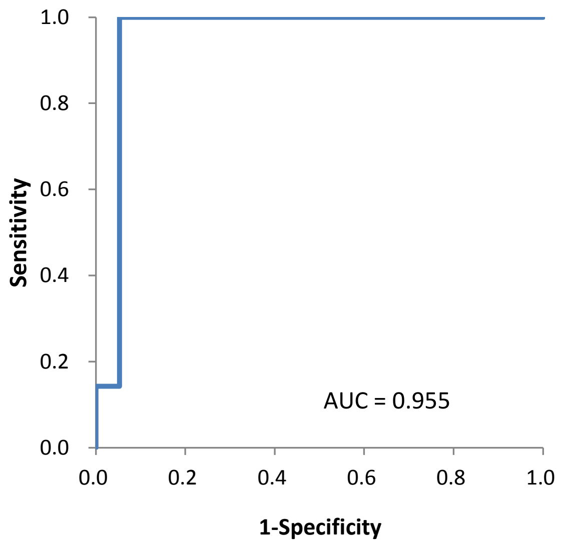

(B)

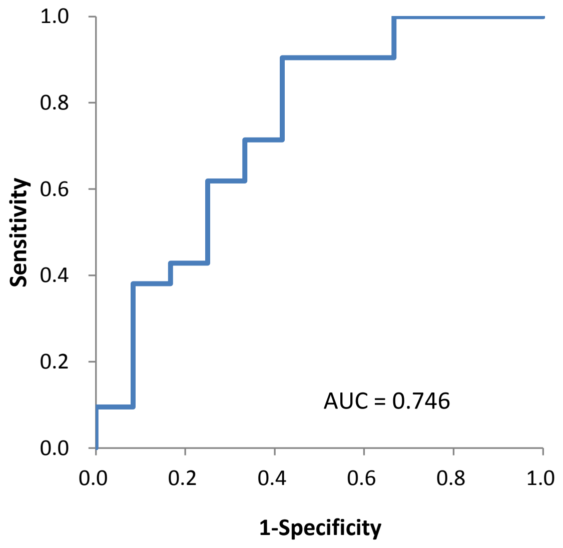

Figure S2 ROC curve analysis for smoking history and alcohol drinking

ROC curve analysis for discrimination between current smoker and never smoker by DNAm rate of *AHRR* (A), habitual and never alcohol drinking by DNAm rate of *HNRNPA1* (B)
